# Association of Mortality and Acute Aortic Events With Ascending Aortic Aneurysm: A Systematic Review and Meta-analysis

**DOI:** 10.1001/jamanetworkopen.2018.1281

**Published:** 2018-08-24

**Authors:** Ming Hao Guo, Jehangir J. Appoo, Richard Saczkowski, Holly N. Smith, Maral Ouzounian, Alexander J. Gregory, Eric J. Herget, Munir Boodhwani

**Affiliations:** 1 Division of Cardiac Surgery, University of Ottawa Heart Institute, Ottawa, Ontario, Canada; 2 Division of Cardiac Surgery, Libin Cardiovascular Institute, University of Calgary, Calgary, Alberta, Canada; 3 Department of Cardiac Sciences, Kelowna General Hospital, Kelowna, British Columbia, Canada; 4 Division of Cardiac Surgery, University of Toronto, Toronto, Ontario, Canada; 5 Department of Anesthesia, University of Calgary, Calgary, Alberta, Canada; 6 Department of Diagnostic Imaging, University of Calgary, Calgary, Alberta, Canada

## Abstract

**Importance:**

The natural history of ascending aortic aneurysm (AsAA) is currently not well characterized.

**Objective:**

To summarize and analyze existing literature on the natural history of AsAA.

**Data Sources:**

A search of Ovid MEDLINE (January 1, 1946, to May 31, 2017) and Embase (January 1, 1974, to May 31, 2017) was conducted.

**Study Selection:**

Studies including patients with AsAA were considered for inclusion; studies were excluded if they considered AsAA, arch, and descending thoracic aneurysm as 1 entity or only included descending aneurysms, patients with heritable or genetic-related aneurysms, patients with replaced bicuspid aortic valves, patients with acute aortic syndrome, or those with mean age less than 16 years. Two independent reviewers identified 20 studies from 7198 unique studies screened.

**Data Extraction and Synthesis:**

Data extraction was performed according to the Meta-analysis of Observational Studies in Epidemiology (MOOSE) reporting guideline; 2 reviewers independently extracted the relevant data. Summary effect measures of the primary outcomes were obtained by logarithmically pooling the data with an inverse variance–weighted random-effects model. Metaregression was performed to assess the relationship between initial aneurysm size, etiology, and the primary outcomes.

**Main Outcomes and Measures:**

The primary composite outcome was incidence of all-cause mortality, aortic dissection, and aortic rupture. Secondary outcomes were growth rate, incidence of proximal aortic dissection or rupture, elective ascending aortic repair, and all-cause mortality.

**Results:**

Twenty studies consisting of 8800 patients (mean [SD] age, 57.75 [9.47] years; 6653 [75.6%] male) with a total follow-up time of 31 823 patient-years were included. The mean AsAA size at enrollment was 42.6 mm (range, 35.5-56.0 mm). The combined effect estimate of annual aneurysm growth rate was 0.61 mm/y (95% CI, 0.23-0.99 mm/y). The pooled incidence of elective aortic surgery was 13.82% (95% CI, 6.45%-21.41%) over a median (interquartile range) follow-up of 4.2 (2.9-15.0) years. The linearized mortality rate was 1.99% per patient-year (95% CI, 0.83%-3.15% per patient-year), and the linearized rate of the composite outcome of all-cause mortality, aortic dissection, and aortic rupture was 2.16% per patient-year (95% CI, 0.79%-3.55% per patient year). There was no significant relationship between year of study completion and the initial aneurysm size and primary outcomes.

**Conclusions and Relevance:**

The growth rate of AsAA is slow and has implications for the interval of imaging follow-up. The data on the risk of dissection, rupture, and death of ascending aortic aneurysm are limited. A randomized clinical trial may be required to understand the benefit of surgical intervention compared with surveillance for patients with moderately dilated ascending aorta.

## Key Points

**Question:**

What are the growth rate and risk of complications in patients with moderately dilated ascending aortas?

**Findings:**

This systematic review and meta-analysis of 20 studies including 8800 patients found that the ascending aorta growth rate was 0.61 mm/y, and the incidence of elective aortic surgery was 13.82%. The linearized mortality rate was 1.99% per patient-year, while the rate of aortic dissection, aortic rupture, and mortality was 2.16% per patient-year.

**Meaning:**

More robust natural history data from prospective studies are needed to better inform clinical decision making in patients with ascending aortic aneurysms.

## Teaser

This systematic review and meta-analysis examines 20 studies including 8800 patients with ascending aortic aneurysm (AsAA) to determine the growth rate of aneurysms; the risk of all-cause mortality, aortic dissection, and aortic rupture; and the incidence of elective surgery in AsAA with tricuspid aortic valve (TAV) or bicuspid aortic valve (BAV).

## Introduction

Ascending aortic aneurysm (AsAA) is generally indolent and asymptomatic until presentation with catastrophic complications of rupture and dissection. When a rupture or dissection occurs, it is fatal in a large proportion of patients prior to hospital presentation. Those who present to the hospital require an emergency surgical procedure that carries a mortality risk of approximately 20%; in contrast, elective ascending aortic replacement carries a relatively low risk of mortality and morbidity.^1^ The goal of elective surgical intervention, therefore, is to provide a survival benefit by replacing the aneurysmal segment of the ascending aorta prior to the occurrence of an acute aortic event. A complete understanding of the natural history of AsAA is critical in determining the risk to benefit ratio of elective aortic surgery.

Aortic diameter is currently the primary variable by which the risk of dissection, rupture, and death is estimated. Existing guidelines recommend surgical repair of asymptomatic AsAA with either tricuspid aortic valve (TAV) or bicuspid aortic valve (BAV) measuring 55 mm or greater in patients without connective tissue disorders, family history, or rapid growth (class I, level of evidence C).^2,3^ These recommendations are largely based on expert consensus and a small number of observational studies.

The purpose of this study is to examine the natural history of AsAA by conducting a systematic review and meta-analysis focusing on end points including all-cause mortality, incidence of ascending aortic dissection or rupture, incidence of elective ascending aortic repair, and aortic growth rate.

## Methods

### Study Selection

An online search of Ovid MEDLINE (January 1, 1946, to May 31, 2017) and Embase (January 1, 1974, to May 31, 2017) was conducted by 1 of us (M.G.) using the Meta-analysis of Observational Studies in Epidemiology (MOOSE) reporting guideline; no search software was used.^4^ The search was conducted with the following search terms: aortic aneurysm* or *aortic aneurysm*, *thoracic/ *or *aortic diseases *or *degenerative aneurysm *or *aorta/thoracic/ *or aortopath* or *bicuspid aortic valve *or *BAV *or bicuspid aortopath* or BAV association* AND *size *or* growth *or medical* or nonsurgical* or *unoperated* AND *natural history* or *survival* or *rupture* or *aortic rupture* or *dissection*. The search was limited to English language publications, resulting in a total of 7196 articles following removal of 2751 duplicates. Two additional studies were identified through a reference search for a total of 7198 studies for initial review. A study was eligible for inclusion if it reported growth rate, rate of dissection or rupture, or all-cause mortality of patients with AsAA. Exclusion criteria included the following: (1) studies that considered all thoracic aortic aneurysms from various regions (ascending, arch, descending thoracic, and thoraco-abdominal) as 1 entity; (2) studies of heritable aortic aneurysms associated with genetic causes, such as Marfan syndrome; (3) studies including nonaneurysmal aortas; (4) studies in which mean patient age was less than 16 years; (5) studies limited to patients with acute aortic syndromes; and (6) editorials, commentaries, case reports, studies with small cohorts (N < 10), and review articles. Reference lists of the included articles were also screened to identify relevant articles. Contact of authors was not required for clarification of the published data. Study selection was done by 2 of us (M.G. and H.N.S.) separately.

### Data Extraction

Two of us (M.G. and H.N.S.) independently extracted relevant data for all studies with a standardized data extraction form; the data extracted were then compared, and all discrepancies were resolved by consensus. Data extraction included demographics, study design, sample size, follow-up, patient risk factors and comorbidities, initial aneurysm diameter, aneurysm growth rate over the follow-up period, incidence of dissection or rupture, size at dissection or rupture, incidence of elective ascending aortic surgery, and all-cause mortality. For studies that were published from the same center for the same patient population, the publication with the longest patient-year follow-up was included in the meta-analysis.

### Statistical Analysis

Studies that did not report on a specific outcome measure were excluded from analysis of that outcome. Summary effect measures for growth rate, incidence of elective aortic repair, aortic dissection or rupture, and all-cause mortality were obtained by logarithmically pooling data with an inverse-variance weighted random-effects model.^5^ The summary effects measures were presented with a 95% confidence interval. When the incidence of an end point was reported as 0, the value was adjusted to an event rate of 1/4 × sample size to permit computation in the random-effects model. To stabilize the variance of a proportion outcome, data underwent the Freeman-Tukey double arcsine transformation.^6^ The incidence of elective aortic repair was presented as a percentage, while all-cause mortality, aortic dissection, and aortic rupture were linearized (percentage per patient-year). A composite linearized outcome of all-cause mortality or aortic dissection or rupture was also evaluated. Metaregression analysis was performed to examine relationships between study-level characteristics, including year of study completion, mean initial aneurysm size, valve type groups, and outcomes. Heterogeneity of the summary effects measures was assessed with the *I*^2^ test and considered present when *I*^2^ was greater than 50%. A 2-sided *P* value of less than .05 was considered statistically significant.

## Results

**Figure 1.**
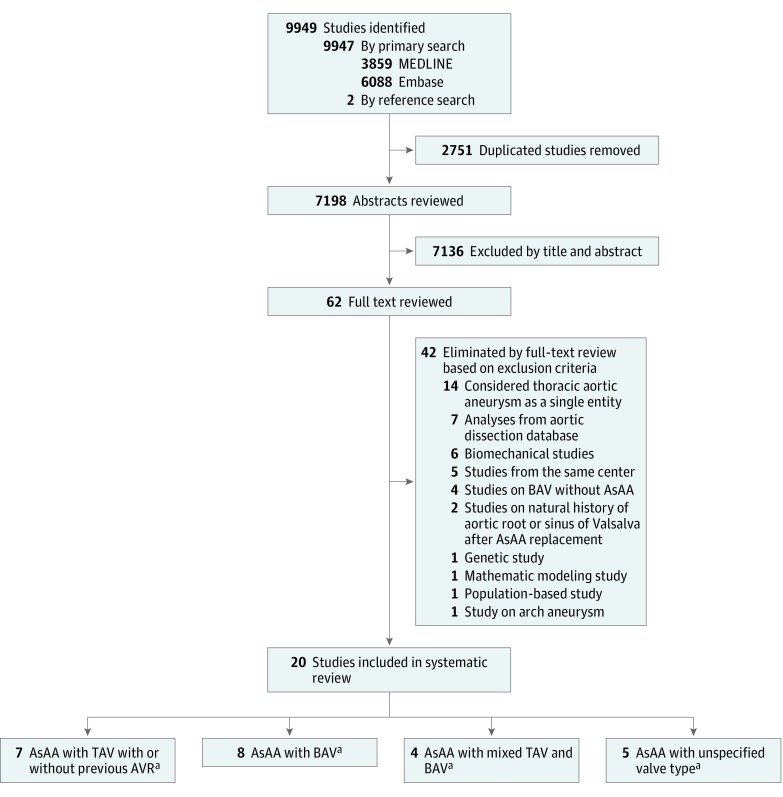
Systematic Review Flowchart ^a^Values overlap because some articles described more than 1 type of valve. AsAA indicates ascending aortic aneurysm; AVR, aortic valve replacement; BAV, bicuspid aortic valve; TAV, tricuspid aortic valve.

The primary search resulted in 7198 studies after removal of duplicates. Of these, 7136 articles were excluded based on title or abstract, and full-text review was completed on 62 studies (Figure 1). The following were then excluded: studies that considered AsAA, arch aneurysm, and descending thoracic aneurysm as a single entity, retrospective analyses of an aortic dissection database, biomechanical or population-based studies that only reported prevalence of the disease rather than its natural history, studies with duplicate cohorts, studies on BAVs without concomitant AsAA, studies on natural history of aortic root or sinus of Valsalva, studies on genetic analysis and mathematical modeling of AsAA, and studies on arch aneurysms only.

### Study Characteristics

Twenty studies published from January 1, 1964, to December 31, 2017, including a total of 8800 patients, were included in the systematic review and data analysis.^7,8,9,10,11,12,13,14,15,16,17,18,19,20,21,22,23,24,25,26^ The total follow-up time was 31 823 patient-years. The studies were divided into 4 groups: AsAA with TAV with or without previous aortic valve replacement, AsAA with native BAV, mixed valve type that included AsAA with either TAV or BAV, and studies that did not specify the valve type. Studies that did not specify valve type did not state explicitly whether aneurysms of hereditary etiologies were excluded. Four studies clearly separated patients with TAV and BAV, and the data were collected separately. The demographic characteristics of the patient population are summarized in the Table. The outcome data of the included articles are summarized in eAppendices 1-4 in the Supplement.

**Table.** Summary of Patient Characteristics of the 20 Studies Included in the Systematic Review

Study	Centers, No.	Study Design	Participants, No.	Male, %	Age, Mean, y	Follow-up, Mean, y	Aneurysm Inclusion Criteria	Method of Imaging	Initial Diameter, Mean (SD), mm
**AsAA With TAV**									
Matsuyama et al,^20^ 2005	1	Retrospective	15	60	57	8.1	>40 mm; post-AVR	CT	NR
La Canna et al,^9^ 2006	1	Retrospective	86	80	61	3.3	40-60 mm	Echo	45.9 (5.1)
Davies et al,^10^ 2007	1	Retrospective	451	63	64.2	5.32	>35 mm	Echo	49.4
Gaudino et al,^21^ 2011	1	Retrospective	93	74	67.1	14.7	50-59 mm; post-AVR	CT	56 (2)
Lee et al,^22^ 2013	1	Retrospective	223	72	59.7	NR	40-55 mm; post-AVR	Echo	43.1 (3)
Detaint et al,^12^ 2014	2	Retrospective	51	78	71	3.6	>40 mm	Echo	44.5 (4)
Kim et al,^16^ 2016	1	Retrospective	4068	78	70.6	3.77	40-55 mm	Echo	41.9 (2.5)
**AsAA With BAV**									
Ferencik and Pape,^23^ 2003	1	Retrospective	68	81	44	3.92	No minimal AsAA size (mean initial aneurysm size 37 mm)	Echo	37 (7)
La Canna et al,^9^ 2006	1	Retrospective	27	96	49	2.96	40-60 mm	Echo	47.3 (5.4)
Davies et al,^10^ 2007	1	Retrospective	70	74	49	5.4	>35 mm	Echo	46.2
Etz et al,^24^ 2010	1	Retrospective	116	NR	NR	4.2	“Dilated” AsAA (mean initial aneurysm size 46 mm)	CT	46 (5)
Michelena et al,^25^ 2011	1	Retrospective	32	69	35	16	>45 mm	Echo	48 (6)
Detaint et al,^12^ 2014	2	Retrospective	353	72	48	3.6	No minimal AsAA size (mean initial aneurysm size 38 mm)	Echo	37.9 (6)
Avadhani et al,^26^ 2015	1	Prospective	90	61	41.8	4.8	No minimal AsAA size (mean initial aneurysm size 36 mm)	Echo	35.5 (5.6)
Kim et al,^16^ 2016	1	Retrospective	586	81	54.4	3.77	40-55 mm	Echo	43.1 (3.2)
**Mixed Valve Type (AsAA With Both TAV and BAV)**									
Geisbusch et al,^11^ 2014	1	Prospective	232	72	63.6	NR	40-49 mm	CT	NR
Gagné-Loranger et al,^14^ 2016	1	Prospective	251	71	65.4	4.3	40-50 mm	CT	NR
Vapnik et al,^15^ 2016	1	Retrospective	628	67	64	3.33	>40 mm	CT/MRI	47 (5)
Park et al,^17^ 2017	1	Retrospective	509	NR	67.2	5.6	>40 mm	CT	NR
**AsAA With Unspecified Valve Type**									
Joyce et al,^7^ 1964	1	Retrospective	26	74	59.3	NR	NR	Fluoroscopy	NR
Masuda et al,^8^ 1992	1	Retrospective	22	72	55	3.4	NR	CT	NR
Andrus et al,^18^ 2003	1	Retrospective	107	69	65.8	2.5	>35 mm; post-AVR	Echo	36 (6)
Bassano et al,^19^ 2005	1	Retrospective	38	82	65	3.5	40-55 mm; post-AVR	Echo	43 (4)
Angeloni et al,^13^ 2015	1	Retrospective	658	62	66.2	NR	NR	Echo	39 (2.5)
Total or mean	NA	NA	8800	75.6	57.75 (9.47)^a^	31 823 patient-years	NA	NA	42.6

Abbreviations: AsAA, ascending aortic aneurysm; AVR, aortic valve replacement; BAV, bicuspid aortic valve; CT, computed tomography; Echo, echocardiography; MRI, magnetic resonance imaging; NA, not applicable; NR, not reported; TAV, tricuspid aortic valve.

^a^ Value given is mean (SD).

There were no randomized clinical trials identified. From the 20 studies, there were 19 single-center studies and 1 dual-center study. Three studies were prospective, while the remaining 17 studies were retrospective. Blinding was not used in any of the studies. The mean length of follow-up ranged from 2.5 to 16.0 years. Eleven studies used echocardiogram as the mode of imaging for follow-up, 8 used computed tomography as the preferred mode of imaging, and 1 used fluoroscopy.

The mean (SD) age of the study patients was 57.75 (9.47) years and 6653 (75.6%) were male. The mean (range) initial aneurysm size among 13 studies was 42.6 mm (35.5-56.0 mm).

### Outcomes

**Figure 2.**
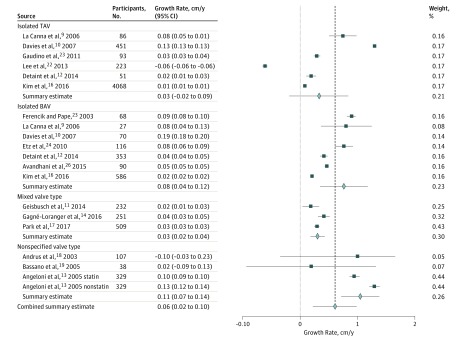
Reported Growth Rate The dashed line is a reference line demarcated at the combined summary estimate and provides a visual reference comparing the subgroup summary estimate with the combined summary estimate. Error bars indicate 95% CIs. BAV indicates bicuspid aortic valve; TAV, tricuspid aortic valve.

Fifteen studies reported the mean annual growth rate, and 3 of these studies reported separate growth rates for AsAA with TAV and AsAA with BAV. For patients with AsAA with TAV, the pooled annual growth rate was 0.34 mm/y (95% CI, −0.18 to 0.85 mm/y; *I*^2^ = 99%). For those with AsAA and native BAV, the pooled annual growth rate was 0.76 mm/y (95% CI, 0.04-1.18 mm/y; *I*^2^ = 99%). The pooled annual growth rate for mixed valve type was 0.31 mm/y (95% CI, 0.19-0.43 mm/y; *I*^2^ = 74%) and was 1.05 mm/y (95% CI, 0.72-1.38 mm/y; *I*^2^ = 91%) for the nonspecified valve type group. The combined effect estimate for annual growth rate for all studies was 0.61 mm/y (95% CI, 0.23-0.99 mm/y; *I*^2^ = 92%) (Figure 2; eAppendix 1 in the Supplement).

**Figure 3.**
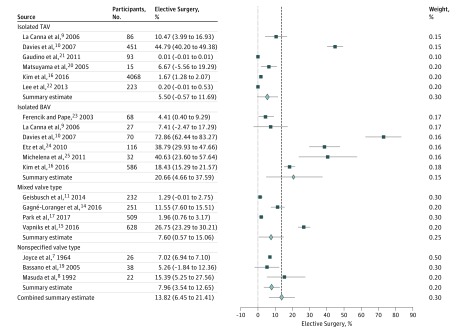
Incidence of Elective Ascending Aortic Surgery The dashed line is a reference line demarcated at the combined summary estimate and provides a visual reference comparing the subgroup summary estimate with the combined summary estimate. Error bars indicate 95% CIs. BAV indicates bicuspid aortic valve; TAV, tricuspid aortic valve.

**Figure 4.**
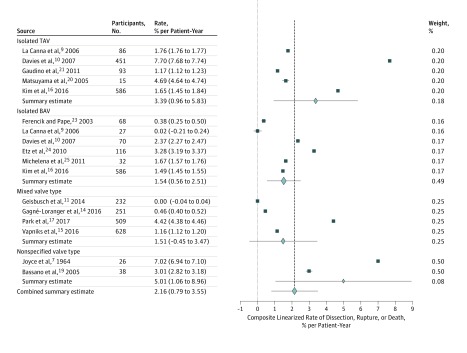
Combined Linearized Rate of Ascending Aortic Dissection, Rupture, or Death The dashed line is a reference line demarcated at the combined summary estimate and provides a visual reference comparing the subgroup summary estimate with the combined summary estimate. Error bars indicate 95% CIs. BAV indicates bicuspid aortic valve; TAV, tricuspid aortic valve.

**Figure 5.**
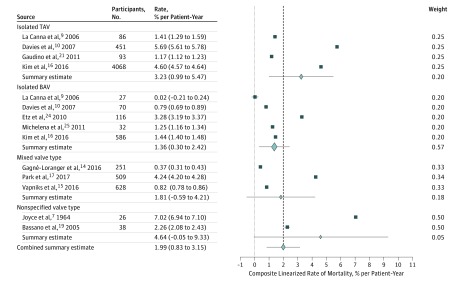
Combined Linearized Rate of All-Cause Mortality The dashed line is a reference line demarcated at the combined summary estimate and provides a visual reference comparing the subgroup summary estimate with the combined summary estimate. Error bars indicate 95% CIs. BAV indicates bicuspid aortic valve; TAV, tricuspid aortic valve.

Fourteen studies reported the incidence of elective aortic surgery during follow up, 12 studies reported incidence of aortic dissection or rupture, and 13 studies reported the incidence of all-cause mortality over follow-up periods that ranged from 2.5 to 16.0 years. The pooled incidence of elective aortic surgery was 13.82% (95% CI, 6.45%-21.41%; *I*^2^ = 56%) over a median (range) follow-up of 4.2 (2.9-15.0) years (Figure 3; eAppendix 2 in the Supplement). For patients with AsAA with TAV, the pooled incidence of elective aortic surgery was 5.50% (95% CI, −0.57% to 11.69%; *I*^2^ = 99%), while for patients with AsAA with BAV, the pooled incidence of elective aortic surgery was 20.66% (95% CI, 4.66%-37.59%; *I*^2^ = 99%). The linearized rate of composite outcome, including aortic dissection, aortic rupture, and all-cause mortality, was 2.16% per patient-year (95% CI, 0.79%-3.55% per patient year; *I*^2^ = 64%), and the linearized rate of composite outcome for patients with AsAA with TAV was 3.39% per patient-year (95% CI, 0.96%-5.83% per patient-year; *I*^2^ = 95%). The linearized all-cause mortality rate was 1.99% per patient-year (95% CI, 0.83%-3.15% per patient-year; *I*^2^ = 84%) (Figure 4 and Figure 5; eAppendices 3 and 4 in the Supplement). The linearized rate of composite outcome for patients with AsAA with TAV was 3.39% per patient-year (95% CI, 0.96%-5.83% per patient-year; *I*^2^ = 95%). Metaregression showed no significant relationship between mean initial aneurysm size and incidence of elective aortic surgery (coefficient, −0.08; 95% CI, −0.22 to 0.05; *P* = .15), incidence of dissection or rupture (coefficient, 0.04; 95% CI, −0.01 to 0.08; *P* = .36), and incidence of all-cause mortality (coefficient, 0.02; 95% CI, −0.72 to 0.12; *P* = .76). Similarly, no significant relationship was found between the year of study completion and the incidence of elective aortic surgery (coefficient, −0.62; 95% CI, −1.58 to 0.39; *P* = .64), incidence of dissection or rupture (coefficient, 0.11; 95% CI, −0.19 to 0.41; *P* = .38), and incidence of all-cause mortality (coefficient, −0.05; 95% CI, −0.65 to 0.54; *P* = .86). Furthermore, using the isolated TAV group as the reference, there were no significant associations between the valve types and the composite outcome (isolated BAV group coefficient, 0.12; 95% CI, −0.05 to 0.31; *P* = .20); mixed valve type group (coefficient, 0.14; 95% CI, −0.06 to 0.36; *P* = .39); and nonspecific valve type group (coefficient, 0.09; 95% CI, −0.08 to 0.28; *P* = .45).

## Discussion

In this study, we sought to explore the natural history of AsAA and to compare outcomes based on valvular phenotype. However, because all except 2 studies followed thoracic aortic management guidelines for ascending aortic repair when patients met criteria for surgical intervention, the true natural history of AsAA across the size spectrum was not examined; instead, we reported on the growth rate; the incidence of aortic repair, dissection, and rupture; and all-cause mortality of AsAA followed in the real-world clinical setting.

The growth rate of the moderately dilated aorta is low, and differences do exist between the different valves types, with large intragroup variation as well (Figure 2). For patients with TAV only, the pooled mean annual growth rate was only 0.34 mm/y, compared with 0.76 mm/y for patients with BAV only. However, this difference was not statistically significant. In both groups, the annual growth rates reported by Davies et al^10^ (1.3 mm/y for the TAV group and 1.9 mm/y for the BAV group) were considerably higher than those reported by other studies in the same group; other studies reported an annual growth rate between −0.6 mm/y and 0.75 mm/y for the TAV group and 0.22 mm/y and 0.9mm/y for the BAV group. The low mean growth rate may have implications for appropriate time interval between imaging studies for follow-up of patients with moderately dilated ascending aorta. Current guidelines suggest annual or biannual imaging for patients with AsAA. Gagné-Loranger et al^14^ argued that contrary to guideline recommendations for follow-up for patients with AsAA, annual or biannual imaging may not be cost-effective. The result of the meta-analysis supports this proposition; however, there are a lack of consistent data that inform the timing in which more frequent follow-up is required. Interestingly, there was a significant difference in the pooled mean annual growth rate between the mixed valve type group and the nonspecified valve type group (0.31 mm/y vs 1.05 mm/y, respectively, *P* < .05). This may represent the inclusion of genetic conditions in the nonspecified valve type group that was not specifically stated within the inclusion and exclusion criteria of the studies, and therefore was not excluded from the systematic review.

There was also significant interstudy variation in all outcomes examined. There was a nonsignificant trend toward higher rate of elective aortic repair in studies reporting on patients with BAV only compared with patients with TAV only (20.66% vs 5.50%, respectively, *P* = .11). However, similar to the data for growth rate, the incidences of elective ascending aortic surgery in the TAV group and BAV group reported by Davies et al^10^ again appeared to be outliers at 44.79% (95% CI, 40.20%-49.38%) and 72.85% (95% CI, 62.44%-83.27%), respectively, when compared with other studies (Figure 3), especially those with similar initial mean aneurysm diameter, such as those reported by Gaudino et al,^21^ La Canna et al,^9^ and Michelena et al.^25^ The linearized rate of composite outcome of 3.39% per patient per year for the TAV group was also much higher compared with other groups, as was the pooled estimate of 2.16% per patient per year (Figure 4). Notably, the rate of composite outcome reported by Davies et al is higher not only when compared with reports with similar initial aneurysm diameter, but also when compared with the rate reported by Joyce et al,^7^ which included patients who were considered nonoperative with an aneurysm size of more than 5.5 cm.

Factors associated with annual growth rate not only varied significantly but were often contradictory between studies; therefore, meaningful statistical analysis evaluating their association with outcomes could not be performed. While 3 studies^8,17,19^ identified baseline aortic diameter as a factor associated with increased growth rate, 4 studies^13,14,18,24^ found that baseline aortic diameter was not associated with aortic dilatation, and 2 studies^12,16^ reported higher annual aortic growth rate with lower baseline aortic diameter. One study^10^ found that aortic stenosis is associated with higher annual aortic dilatation, while another^17^ found aortic regurgitation to be associated with higher growth rate. Another study^26^ determined that valvular dysfunction is not associated with faster aortic dilatation. Renal failure and female sex were identified as being associated with faster annual growth rate in 2 separate studies,^14,16^ while anticoagulation and statin medication use were found be associated with a lower growth rate.^11,13^ In addition, factors associated with increased risk of dissection and rupture were poorly reported. Coady et al^27^ and Vapnik et al^15^ demonstrated that size was the most important factor associated with acute dissection or rupture; however, most other studies did not report aneurysm size at aortic dissection or rupture, and comparison was therefore not possible.

Current guidelines for surgical thresholds for AsAA are based largely on expert consensus and retrospective observational studies with inconsistent data. In contrast, treatment guidelines for abdominal aortic aneurysm (AAA) are founded on extensive data and are widely accepted by the surgical community. Multiple large, multicenter randomized clinical trials that included more than 200 000 patients established the natural history of small AAA (<55 mm) as well as proper surveillance for patients with this life-threatening disease.^28,29,30,31,32^ By comparing long-term outcomes of patients randomized to either the surveillance group (surgery if aneurysm size is >55 mm or growth rate is >10 mm/y) or the early surgery group, the trials have shown that for AAA smaller than 55 mm, the risk of early surgery outweighs the risk of rupture during surveillance and that women have a higher risk of rupture than men. On the other hand, the natural history of large AAA (>55 mm) has also been explored in prospective studies in which patients with AAA larger than 55 mm who were not suitable surgical candidates were followed.^33,34,35^ Although most studies confirmed that large AAA has a higher rupture rate than small AAA, controversies exist on whether the difference in rupture rate reflects the difference between patients at high operative risk with severe comorbidities and the healthier patients randomized in clinical trials.^35^ Nonetheless, in contrast to the literature in the management of AAA, clinical trials that guide practice for AsAA do not currently exist and represent an important gap in knowledge for the condition’s management.

### Limitations

This study has a number of limitations. First, this is a meta-analysis of studies that included a heterogenous population with different initial mean aortic diameters, variable lengths of follow-up, and different imaging methods, which may introduce variability into the combined effect estimates. Second, most studies focused on a specific outcome of the natural history of AsAA, and information on all outcomes was not available in all studies. There were insufficient data available to perform analysis for factors associated with growth rate, the rate of composite outcome, or aortic size at acute aortic events. Third, more than half of the articles included were published prior to 2010 and therefore may not represent contemporary medical management of this disease. Fourth, there might be confounders that are not adjusted for in the studies included.

## Conclusions

The growth rate of the moderately dilated ascending aorta is low. The risk of dissection, rupture, and death also remains low. However, these results require cautious interpretation as a large number of patients in the studies who met guideline criteria for intervention underwent elective aortic surgery. More robust natural history data from prospective studies or randomized clinical trials are necessary to better inform clinical decision making in patients with ascending aortic disease.

## Notes

Supplement. **eAppendix 1. **Mean Initial Aneurysm Diameter and Growth Rate From the Included Articles **eAppendix 2.** Incidences of Aortic Dissection or Rupture From the Published Articles **eAppendix 3.** Incidences of Elective Aortic Surgery From the Published Articles **eAppendix 4.** All-Cause Mortality From the Published Articles
