# Supplementary material for: Association of Mortality and Acute Aortic Events With Ascending Aortic Aneurysm: A Systematic Review and Meta-analysis
Source: JAMA Netw Open. 2018 Aug 24;1(4):e181281. doi: 10.1001/jamanetworkopen.2018.1281 (PMC6324275; doi:10.1001/jamanetworkopen.2018.1281)
Supplement: Supplement. — eAppendix 1. Mean Initial Aneurysm Diameter and Growth Rate From the Included Articles eAppendix 2. Incidences of Aortic Dissection or Rupture From the Published Articles eAppendix 3. Incidences of Elective Aortic Surgery From the Published Articles eAppendix 4. All-Cause Mortality From the Published Articles [file jamanetwopen-1-e181281-s001.pdf]

Supplementary Online Content

Guo M, Appoo JJ, Saczkowski R, et al. Association of mortality and acute aortic events with ascending aortic aneurysm: a systematic review and meta-analysis. *JAMA Netw Open*. 2018;1(4):e181281. doi:10.1001/jamanetworkopen.2018.1281

eAppendix 1. Mean Initial Aneurysm Diameter and Growth Rate From the Included Articles
eAppendix 2. Incidences of Aortic Dissection or Rupture From the Published Articles
eAppendix 3. Incidences of Elective Aortic Surgery From the Published Articles
eAppendix 4. All-Cause Mortality From the Published Articles

This supplementary material has been provided by the authors to give readers additional information about their work.

© 2018 Guo M et al. *JAMA Network Open.*

eAppendix 1. Mean initial aneurysm diameter and growth rate from the included articles. *TAV = tricuspid aortic valve; BAV = bicuspid aortci valve; FU = Follow-up*

Author	Year	MEAN INITIAL ANEURYSM DIAMETER (cm)	GROWTH RATE			
			Mean Growth rate (cm/yr)	Sample Size	Mean FU Time	Median FU Time
**ISOLATED TAVs**						
La Cana, G.	2006	4.59 ± 0.51	0.075 ± 0.11	86	3.31 ± 2.33	-
Davies, R. R.	2007	4.94	0.13 ± 0.02	451	3.52	-
Gaudino M.	2011	5.6 ± 0.2	0.03 ± 0.02	64	14.7 ± 4.8	-
Detaint, D.	2014	4.45 ± 0.4	0.02 ± 0.03	51	3.6 ± 1.2	-
Matsuyama, K.	2005	-	-	-	-	-
Lee SH	2013	4.31 ± 0.3	-0.06 (IQR: -0.17, 0.19; median)	223	-	2.08 (IQR 1.05, 3.99)
Kim JB	2016	4.19 ± 0.25	0.009 ± 0.004	1414	-	3.91 (IQR: 2.14, 5)
**ISOLATED BAVs**						
Ferencik, M.	2003	3.7 ± 0.7	0.09 ± 0.03	68	3.91 ± 2.25	-
La Cana, G.	2006	4.73 ± 0.54	0.081 ± 0.11	27	2.96 ± 2.17	-
Davies, R.R.	2007	4.62	0.19 ± 0.06	70	3.6	-
Etz, C.D.	2010	4.6 ± 0.5	0.077	116	4.2 ± 2.9	-
Michelena, H.I.	2011	4.8 ± 0.6	-	-	-	-
Detaint, D.	2014	3.79 ± 0.6	0.042 ± 0.06	353	3.6 ± 1.2	-
Avadhani, S.A.	2015	3.55 ± 0.56	0.047 ± 0.005	90	4.8	-
Kim, JB.	2016	4.31 ± 0.32	0.022 ± 0.008	586	-	3.91 (IQR: 2.14, 5)
**MIXED TAVs + BAVs**						
Geisbusch, S.	2014	-	0.019 ± 0.09	166	4.19	-
Gagnes-Loranger, M.	2016	-	0.042 ± 0.082	251	4.3 ± 2.5	-
Vapniks, JS.	2016	4.7 ± 0.5	-	-	-	-
Park KH	2017		0.03 ± 0.05	509	4.3 ± 2.4	5.3 (IQR: 3.4, 7.6)
**UNSPECIFIED**						
Joyce, J.W.	1964		-	-	-	-
Masuda, Y.	1992		-	-	-	-
Andrus, B.W.	2003	3.6 ± 0.6	-0.10 ± 0.70	107	2.8	-
Bassano, C.	2005	4.3 ± 0.4	0.02 ± 0.34	38	3.5 ± 2.3	-
Angeloni, E.	2015	3.89 ± 0.25	Statin: 0.095	329	2.88 ± 0.55	-
		3.91 ± 0.26	non-Statin: 0.13	329	2.88 ± 0.55	

© 2018 Guo M et al. *JAMA Network Open.*

eAppendix 2. Incidences of aortic dissection or rupture from the published articles. *TAV = tricuspid aortic valve; BAV = bicuspid aortic valve; AD = aortic dissection; R = rupture; UD = unknown death; FU = Follow-up*

Author	Year	INCIDENCES OF AD OR R							
		Reported Number of Events					Sample Size	Mean FU Time	Median FU Time
		AD	R	AD or R	UD	AD/R/UD			
**ISOLATED TAVs**									
La Cana, G.	2006	1	0	1	2	3	86	3.31 ± 2.33	-
Davies, R. R.	2007	38	15	47	-	116	451	5.31	-
Gaudino M.	2011	0	0	0	0	0	93	14.7 ± 4.8	-
Detaint, D.	2014	-	-	-	-	-	-	-	-
Matsuyama, K.	2005	-	-	2	-	-	15	8.1	-
Lee SH	2013	1	-			-	223	-	3.63 (IQR: 2.04, 5.73)
Kim JB	2016	12	1	13	-	-	4068	3.77	3.34 (IQR: 1.36, 5)
**ISOLATED BAVs**									
Ferencik, M.	2003	1	-	1	-	-	68	3.91 ± 2.25	-
La Cana, G.	2006	0	0	0	0	0	27	2.96 ± 2.17	-
Davies, R.R.	2007	4	2	6	-	6	70	5.43	-
Etz, C.D.	2010	0	0	0	4	4	116	4.2 ± 2.9	-
Michelena, H.I.	2011	2	0	2	0	2	32	15 ± 6	-
Detaint, D.	2014	-	-	-	-	-	-	-	-
Avadhani, S.A.	2015	-	-	-	-	-	-	-	-
Kim, JB.	2016	1	0	1	-	-	586	3.77	3.34 (IQR: 1.36, 5)
**MIXED TAVs + BAVs**									
Geisbusch, S.	2014	0	0	0	0	0	232	4.19	-
Gagnes-Loranger, M.	2016	1	0	1	0	1	251	4.3 ± 2.5	-
Vapniks, JS.	2016	-	-	7	-	-	628	-	3.33
Park KH	2017	5	-	5	-	-	509	5.6 ± 2.6	-
**UNSPECIFIED**									
Joyce, J.W.	1964	-	-	-	-	-	-	-	-
Masuda, Y.	1992	-	2	-	-	-	22	3.4	-
Andrus, B.W.	2003	-	-	-	-	-	-	-	-
Bassano, C.	2005	1	0	1	2	4	38	3.5 ± 2.3	-
Angeloni, E.	2015	-	-	-	-	-	-	-	-

© 2018 Guo M et al. *JAMA Network Open.*

eAppendix 3. Incidences of elective aortic surgery from the published articles. *TAV = tricuspid aortic valve; BAV = bicuspid aortic valve; No. = number; FU = Follow-up*

Author	Year	INCIDENCE OF ELECTIVE AORTIC SURGERY			
		No. of Events	Total Sample Size	Mean FU Time	Median FU Time
**ISOLATED TAVs**					
La Cana, G.	2006	9	86	3.31 ± 2.33	-
Davies, R. R.	2007	202	451	5.31	-
Gaudino M.	2011	0	93	14.7 ± 4.8	-
Detaint, D.	2014	-	-	-	-
Matsuyama, K.	2005	1	15	8.1	-
Lee SH	2013	0	223	-	3.63 (IQR: 2.04, 5.73)
Kim JB	2016	68	4068	3.77	3.34 (IQR: 1.36, 5)
**ISOLATED BAVs**					
Ferencik, M.	2003	3	68	3.91 ± 2.25	-
La Cana, G.	2006	2	27	2.96 ± 2.17	-
Davies, R.R.	2007	51	70	5.43	-
Etz, C.D.	2010	45	116	4.2 ± 2.9	-
Michelena, H.I.	2011	13	32	15 ± 6	-
Detaint, D.	2014	-	-	-	-
Avadhani, S.A.	2015	-	-	-	-
Kim, JB.	2016	108	586	3.77	3.34 (IQR: 1.36, 5)
**MIXED TAVs + BAVs**					
Geisbusch, S.	2014	3	232	4.19	-
Gagnes-Loranger, M.	2016	29	251	4.3 ± 2.5	-
Vapniks, JS.	2016	168	628	-	3.33
Park KH	2017	10	509	5.6 ± 2.6	-
**UNSPECIFIED**					
Joyce, J.W.	1964	-	-	-	-
Masuda, Y.	1992	5	22	3.4	-
Andrus, B.W.	2003	-	-	-	-
Bassano, C.	2005	2	38	3.5 ± 2.3	-
Angeloni E.	2015	-	-	-	-

© 2018 Guo M et al. *JAMA Network Open.*

eAppendix 4. All-cause mortality from the published articles. *TAV = tricuspid aortic valve; BAV = bicuspid aortic valve; No. = number; FU = Follow-up*

Author	Year	ALL-CAUSE MORTALITY			
		No. of Event (Death)	Sample Size	Mean FU Time	Median FU Time
**ISOLATED TAVs**					
La Cana, G.	2006	5	86	3.31 ± 2.33	-
Davies, R. R.	2007	137	451	5.31	-
Gaudino M.	2011	16	93	14.7 ± 4.8	-
Detaint, D.	2014	-	-	-	-
Matsuyama, K.	2005	-	-	-	-
Lee SH	2013	-	-	-	-
Kim JB	2016	706	4068	3.77	3.34 (IQR: 1.36, 5)
**ISOLATED BAVs**					
Ferencik, M.	2003	-	-	-	-
La Cana, G.	2006	0	27	2.96 ± 2.17	-
Davies, R.R.	2007	3	70	5.43	-
Etz, C.D.	2010	16	116	4.2 ± 2.9	-
Michelena, H.I.	2011	6	32	15 ± 6	-
Detaint, D.	2014	-	-	-	-
Avadhani, S.A.	2015	-	-	-	-
Kim, JB.	2016	32	586	3.77	3.34 (IQR: 1.36, 5)
**MIXED TAVs + BAVs**					
Geisbusch, S.	2014	-	-	-	-
Gagnes-Loranger, M.	2016	4	251	4.3 ± 2.5	-
Vapniks, JS.	2016	17	628	-	3.33
Park KH	2017	121	509	5.6 ± 2.6	-
**UNSPECIFIED**					
Joyce, J.W.	1964	40	57	10	-
Masuda, Y.	1992	-	-	-	-
Andrus, B.W.	2003	-	-	-	-
Bassano, C.	2005	3	38	3.5 ± 2.3	-
Angeloni E.	2015	-	-	-	-

© 2018 Guo M et al. *JAMA Network Open.*
